# Efficacy and Safety of Deep Brain Stimulation in the Treatment of Parkinson’s Disease: A Systematic Review and Meta-analysis of Randomized Controlled Trials

**DOI:** 10.7759/cureus.3474

**Published:** 2018-10-22

**Authors:** Sosipatros Bratsos, Dimitrios Karponis, Sohag N Saleh

**Affiliations:** 1 Internal Medicine, Imperial College London, London, GBR; 2 Orthopaedics, Imperial College London, London, GBR; 3 Pharmacology, Imperial College London, London, GBR

**Keywords:** medical therapy, parkinson’s disease, systematic review, meta-analysis, deep brain stimulation (dbs)

## Abstract

Deep brain stimulation (DBS) is a neurosurgical procedure indicated for patients with advanced Parkinson’s disease (PD). Whether similar benefits may be realized by patients with early PD, however, is currently unclear, especially given the potential risks of the procedure. This systematic review and meta-analysis aimed to investigate the relative efficacy and safety of DBS in comparison to best medical therapy (BMT) in the treatment of PD. It also aimed to compare the efficacy of DBS between patients with early and advanced PD.

A systematic search was performed in Medline, Embase, and Cochrane Central Register of Controlled Trials (CENTRAL). Randomized controlled trials (RCTs) comparing DBS to BMT in PD patients were included. Outcome measures were impairment/disability using the Unified Parkinson’s Disease Rating Scale (UPDRS), quality of life (QoL) using the Parkinson's Disease Questionnaire (PDQ-39), levodopa equivalent dose (LED) reduction, and rates of serious adverse events (SAE).

Eight eligible RCTs (n = 1,189) were included in the meta-analysis, two of which recruited early PD patients. Regarding efficacy outcomes, there were significant improvements in UPDRS, PDQ-39, and LED scores in favour of DBS (P < 0.00001). There was a significantly greater reduction of LED in patients with early PD (P < 0.00001), but no other differences between early and advanced PD patients were found. The risk of a patient experiencing an SAE was significantly higher in the DBS group (P = 0.005), as was the total number of SAEs (P < 0.00188).

Overall, DBS was superior to BMT at improving impairment/disability, QoL, and reducing medication doses, but these benefits need to be weighed against the higher risk of SAEs. There was insufficient evidence to determine the impact of the PD stage on the efficacy of DBS.

## Introduction and background

Parkinson’s disease

Parkinson’s disease (PD) is a progressive and debilitating neurodegenerative disorder that affects 0.1 - 0.2% of the population at any time and 1% of the population over 60 years old, with the prevalence increasing in an age-dependent manner [1]. Clinically, it is characterised by motor, autonomic, and neurocognitive impairment. The cardinal motor signs are tremor, rigidity, and bradykinesia [2] and the autonomic features include constipation and orthostatic hypotension, while neurocognitive dysfunction can manifest itself as depression or sleep disorders [3]. Key pathophysiological findings of PD are the loss of dopaminergic neurons in the substantia nigra and α-synuclein-containing Lewy bodies, while its aetiology implicates both environmental and genetic (e.g., SNCA and LRRK2 mutations) factors [4].

Currently, the management of PD focuses on drug therapy and primarily on levodopa, which is regarded as the most effective treatment [5]. However, long-term levodopa use is associated with several side-effects, such as dyskinesias and on-off fluctuations, that present after an initial honeymoon period of sustained response [6]. Other agents that are commonly used to treat motor complications, e.g. dopamine agonists, are also linked to a poor side-effect profile; thus, the patient's quality of life (QoL) tends to progressively deteriorate [7]. Hence, novel therapeutic approaches are needed.

Deep brain stimulation: procedure

Deep brain stimulation (DBS) is the United Kingdom National Institute for Health and Care Excellence (NICE) and United States Federal Drug Administration (FDA)-approved surgical procedure for advanced PD, but it is being increasingly used for earlier stages of PD. It involves the stereotactic implantation of an electrode in specific brain structures [8]. The electrode is connected to a pulse generator, which is implanted subcutaneously and transmits high-frequency electrical impulses to the target area. The DBS settings are externally programmed via a hand-held device using an electro-modulator [9].

Deep brain stimulation: mechanism of action

Several hypotheses have been proposed about how DBS works, but its exact mechanism of action remains elusive [10]. Firstly, DBS is thought to act by shifting the low frequency (15 - 30 Hz) oscillatory activity observed in PD to a higher frequency, thus increasing the firing rate of the stimulated nucleus (commonly, the subthalamic nucleus (STN), globus pallidus internus (GPi), or the caudal zona incerta (cZi)) and activating adjacent fibre tracts that modulate the entire basal ganglia-thalamocortical network [11]. Moreover, it has been suggested that DBS changes the firing rate and pattern of individual neurons in the basal ganglia, replacing the irregular, aberrant electrical activity of the stimulated nucleus with a stimulus-induced regular pattern that prevents the spread of pathologic firing, leading to improved processing of sensorimotor information and, therefore, the amelioration of motor symptoms [12-15].

Current literature

Systematic reviews and meta-analyses of randomized controlled trials (RCTs) comparing DBS to the best medical therapy (BMT) are limited. Perestelo-Pérez et al. [16] showed that DBS was superior to BMT for improving motor control, QoL, and medication doses in PD patients. Most publications, however, have either compared STN-DBS to GPi-DBS or one of them to BMT. For example, Tan et al. [17] showed that STN- and GPi-DBS are similarly effective at treating PD symptoms, while Xie et al. [18] added that DBS in either site is more effective than BMT and suggested that stimulation of each site produced benefits in different symptom domains. Nevertheless, systematic reviews have narrowed their focus on DBS of the STN or GPi only, despite evidence that DBS of other target structures (e.g., cZi) has been investigated. In addition, the impact of PD stage (i.e., early/advanced) on the effectiveness of DBS has yet to be evaluated, and finally, no meta-analysis comparing the safety profile of DBS and BMT has been conducted.

Aims, objectives, and hypothesis

This review aimed to provide a comprehensive overview and quantitative summary of RCTs that compared DBS (STN, GPi, or other) to BMT in terms of efficacy and safety in PD patients. It also aimed to compare the efficacy of DBS in early vs. advanced PD patients. It was hypothesized that DBS would have superior efficacy [16, 18] and inferior safety profile [19-20] than BMT. Thus, the objectives of this review were to perform a thorough systematic search of the available evidence regarding DBS as a treatment for PD, select eligible studies according to pre-determined inclusion and exclusion criteria, extract appropriate outcome variables and protocol information from the available evidence, and conduct a meta-analysis of the extracted data to test the hypothesis stated above.

## Review

Criteria for considering studies for this review

Types of Studies

Randomised controlled trials (RCTs) with parallel-group designs were included.

Types of Participants

Adult participants (> 18) with Parkinson's disease (PD) who have been diagnosed by the UK Parkinson's Disease Brain Bank criteria [21], were included, regardless of medication, duration of illness, the presence of motor fluctuations, duration of treatment, or level of initial impairment.

Types of Interventions

DBS of any kind (i.e., unilateral or bilateral; any target area) was compared to BMT.

Primary Outcomes - Efficacy

• Impairment/disability, as measured by the UPDRS (Unified Parkinson’s disease rating scale): I. Mental status, behaviour, mood; II. Activities of daily living (ADLs); III. Motor function; and IV. Complications from therapy [22]

• Health-related QoL, according to the PDQ-39 (Parkinson’s disease questionnaire) [23]

• Levodopa-equivalent dose (LED) reduction

Secondary Outcomes - Safety

• Patients with serious adverse events (SAEs)

• Total number of SAEs

Search methods for identification of studies

Electronic Searches

An electronic search was carried out on three databases:

• Embase Classic and Embase: 1947 – May 05, 2018

• Medline: 1946 – May 05, 2018 

• Cochrane Central Register of Controlled Trials (CENTRAL): all years – May 05, 2018  

The search strategy was developed using a combination of subject headings and free-text terms for “Parkinson’s disease” and “deep brain stimulation”, as well as their synonyms, related terms, and variant spellings in order to include a more comprehensive literature search. No time limit was set in an effort not to miss potentially eligible articles. Sensitivity-maximising search strategies for RCTs were used for both Medline and Embase, according to the Cochrane Handbook for Systematic Reviews of Interventions [24]. In CENTRAL, an RCT filter was not necessary, as all records are correctly indexed.

Data collection and analysis

Selection of Studies

Search results were exported to Covidence systematic review software (Veritas Health Innovation, Melbourne, Australia) [25]. Duplicates were removed before the titles, abstracts of the records identified through the electronic searches were screened, and clearly irrelevant references, according to the inclusion/exclusion criteria (Table 1), were eliminated. The full-text of the remaining studies was retrieved and relevant studies for inclusion were checked according to the inclusion/exclusion criteria.

**Table 1:** Study Inclusion and Exclusion Criteria Inclusion and exclusion criteria for the systematic review and meta-analysis are shown. CENTRAL: Cochrane Central Register of Controlled Trials; DBS: deep brain stimulation; GPi: globus pallidus internus; LED: levodopa equivalent dose; N/A: not applicable; PDQ-39: Parkinson’s disease questionnaire; STN: subthalamic nucleus; UPDRS: Unified Parkinson’s disease rating scale

Parameter		Inclusion Criteria		Exclusion Criteria
Time period		Embase Classic and Embase: 1947–May 05, 2018		N/A
		Medline: 1946–May 05, 2018		
		CENTRAL: all years–May 05, 2018		
Study design		Prospective		Retrospective
		Randomized controlled trial		Uncontrolled or non-randomized
		English publication		Conference proceedings or abstracts
Participants		Humans		Animals
		Parkinson's disease diagnosis		Non-Parkinson's disease pathology
Intervention		Deep brain stimulation		Transcranial direct current stimulation
Comparison		Medical therapy		STN-DBS vs GPi-DBS
Outcomes		UPDRS		Neuropsychological changes
		PDQ-39		
		LED		Data not extractable
		Adverse events		

Data extraction and management

Trial and outcome data from the selected trials were extracted according to pre-specified checklists, as shown below:

• Participants (country, number of participants, age, gender, stage of PD as assessed by Hoehn and Yahr (H-Y) at study entry [26], 'on'/'off' state of dopaminergic medication, inclusion/exclusion criteria)

• Comparison (details of interventions in treatment and control groups, duration of treatment)

• Outcomes (data were extracted as means with standard deviations; if means were not provided, baseline means were subtracted from outcome means; if standard deviations (SD) were not provided, they were imputed using a correlation coefficient [24] or were calculated from standard errors, confidence interval (CI), or other statistical indices)

Assessment of risk of bias in included studies

The risk of bias in the included trials was assessed at both study and domain levels, according to the Cochrane Collaboration’s tool for assessing risk of bias [24] and was graphically presented using Review Manager (RevMan) 5.3 (The Nordic Cochrane Centre, Copenhagen, The Cochrane Collaboration, 2014) [27]. The risk of bias was categorised as ‘low’, ‘high’ or ‘unclear’ for the following domains:

• Random sequence generation

• Allocation concealment

• Blinding of participants and personnel

• Blinding of outcome assessment

• Selective outcome reporting

• Incomplete outcome data

• Other bias

Measures of treatment effect

For UPDRS, PDQ-39, and LED (continuous data), the mean (SD) change from baseline was used to generate effect estimates. A summary estimate of the mean difference (MD) with 95% CI was calculated. For SAE, two comparisons between the two groups were made: 1) the risk of a patient experiencing an SAE (dichotomous data), where risk ratios (RRs) with 95% CIs were calculated, and 2) the total number of SAE, where the data were initially tested for normality using the Kolmogorov-Smirnov test in order to determine whether the independent-samples-t-test or the Mann–Whitney U test should be performed. RevMan was used for all meta-analyses and online calculators [28-29] were used for the other statistical comparisons. P < 0.05 was considered significant.

Dealing with missing data

Attempts were made to contact four corresponding authors to request missing or unreported data, all without success.

Assessment of heterogeneity

The I² statistic was used to assess heterogeneity, as it emphasizes the effect of heterogeneity, rather than merely reporting its presence [24]. In cases where I² was greater than 50%, significant heterogeneity was assumed.

Assessment of publication biases

The presence of publication bias was assessed by visual inspection of funnel plots, if appropriate.

Data synthesis

Given the potential clinical or methodologic heterogeneity between studies (variation in PD stage, types of DBS/target areas, follow-up period, use of blinding, and concealment allocation), a random-effects model was used, which yields a more conservative estimate of the pooled effect and would not violate the preconditions of a fixed-effect model.

Investigation of heterogeneity

Subgroup Analysis

If sufficient data were available, a subgroup analysis based on the stage of PD (early/advanced) was conducted (RevMan [27]) for UPDRS, PDQ-39, and LED.

Sensitivity Analysis

One study was excluded at a time and the impact of removing each of the studies was evaluated on between-study heterogeneity in the primary and secondary outcomes [30].

Results of the search

**Figure 1:**
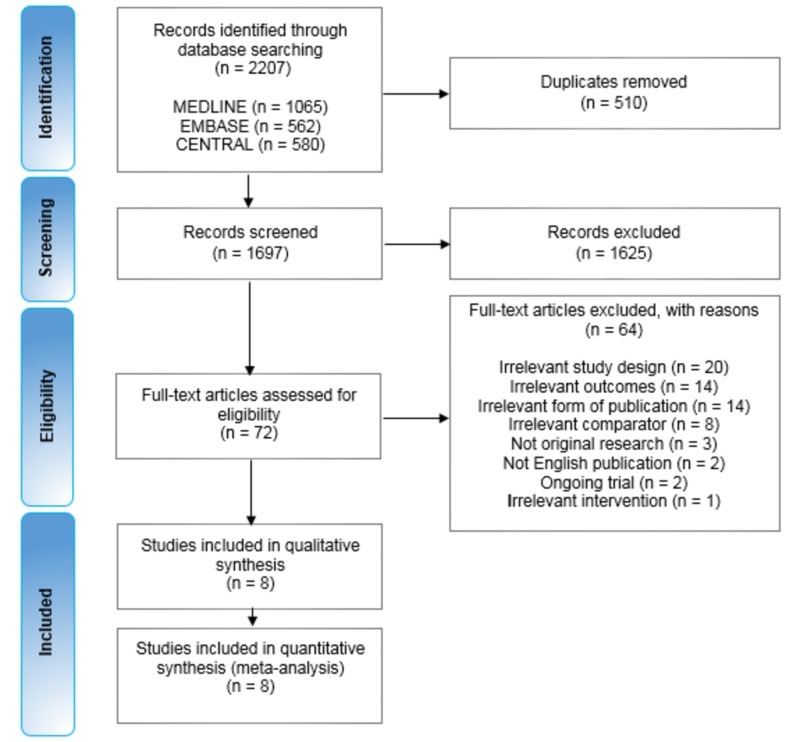
Study flow diagram The top boxes show the number of records identified in each of the three databases, followed by the total number of records, before and after duplicates were removed. The number of records screened and excluded on the basis of the title and abstract follows. Below this, is the number of full-text articles assessed for eligibility, and the number of those excluded, with reasons as listed. Eight studies were included in the qualitative and quantitative synthesis (meta-analysis). CENTRAL: Cochrane Central Register of Controlled Trials

A total of 2,207 records across three databases were identified. After duplicates were removed, 1,697 records were screened (abstract and title) and 72 full-text articles were assessed for eligibility. After further assessment, it was determined that the review inclusion criteria were met by eight studies (Figure 1).

Included studies

Eight studies [31-38] (range (n) = 19-366 patients) were included with a total number of 1,189 participants. All studies were parallel-group RCTs, with two of them, Deuschl et al. and Schüpbach et al. [34, 36], utilising a randomised-pair design, whereas in the Williams et al. study [33], this was left to the choice of the participating centres. Although DBS in the experimental group was performed bilaterally in all studies, the specific brain region in which it was performed varied. In five studies, it involved the STN, but half of the experimental group (n = 61) in the Weaver et al. [38] and (n = 4) participants in the Williams et al. [33] studies received stimulation of the GPi, while in the study by Blomstedt et al. [31] DBS was performed in the cZi. Also, a novel wireless DBS programming system and a constant-current DBS device were utilised by Li et al. [32] and Okun et al. [37], respectively. The control group in all studies received medical therapy. However, in the Okun et al. [37] and Li et al. [32] studies, the DBS device was implanted in all participants; however, in the control group, it was not activated until three months after implantation. Therefore, data for these two studies were extracted up to the three-month endpoint. The follow-up period for the remaining studies was six [31, 34, 38], 12 [33], 18 [36], and 24 [35] months. The mean age of the participants was 59.6 years, excluding Schüpbach et al. [36] and Schuepbach et al. [35] (who recruited patients with early symptoms of PD) with mean ages of 48.5 and 52.5 years, respectively. In terms of outcome measures, UPDRS-I was reported in five studies [31, 33, 35, 37-38]; UPDRS-II (on or off) was observed in seven [32-38] or four studies [33-36], respectively; UPDRS-III (on or off) was reported in seven studies [31-35, 37-38]; UPDRS-IV was reported in five studies [32-33, 35, 37-38]. Moreover, PDQ-39 and LED (pre- and post-surgery) were reported in five [31, 33-35, 38] and six [31-32, 34-35, 37-38] studies, respectively. Finally, SAEs were reported in six studies [31, 33-35, 37-38]. Study characteristics and patient demographics are summarised below (Tables 2, 3).

**Table 2:** Summary of Characteristics of Included Studies Randomized patients, n, refers to the total number of randomized patients in both experimental and control groups; Settings refer to the stimulation parameters (amplitude, pulse width duration, frequency); “on/off” refer to patients being on or off medication. BMT: best medical therapy; cZi: caudal zona incerta; DBS: deep brain stimulation; Gpi: globus pallidus internus; Hz: hertz; LED: levodopa equivalent dose; PDQ-39: Parkinson’s disease questionnaire; RCT: randomized controlled trial; SAE: serious adverse effects; STN: subthalamic nucleus; UPDRS: Unified Parkinson’s Disease Ranking Scale; μs: microseconds; V: voltage

Study		Randomized patients, n	Design	Intervention (settings)	Control	Follow-up (months)	Outcomes
Blomstedt et al. [31]		19	RCT	DBS-cZi (2.0-3.5 V, 60 μs, 130 Hz)	BMT	6	UPDRS-III (ON/OFF), PDQ-39, LED, SAE
Deuschl et al. [34]		78	RCT	DBS-STN (variable V, 60 μs, 130 Hz)	BMT	6	UPDRS-II (ON/OFF), UPDRS-III (ON/OFF), PDQ-39, LED, SAE
Li et al. [32]		64	RCT	Wireless DBS-STN (0-10 V, 60-960 ms, 1-1,600 Hz)	BMT	3	UPDRS-I, UPDRS-II (ON), UPDRS-III (ON/OFF), UPDRS-IV, LED
Okun et al. [37]		136	RCT	DBS-STN (not reported)	BMT	3	UPDRS-I, UPDRS-II (ON), UPDRS-III (ON/OFF), UPDRS-IV, PDQ-39, LED, SAE
Schuepbach et al. [35]		251	RCT	DBS-STN (variable V, 60 μs, 130 Hz)	BMT	24	UPDRS-I, UPDRS-II (ON/OFF), UPDRS-III (ON/OFF), UPDRS-IV, PDQ-39, LED, SAE
Schüpbach et al. [36]		20	RCT	DBS-STN (3.1 V, 69 μs, 167 Hz)	BMT	18	UPDRS-II (ON/OFF)
Weaver et al. [38]		255	RCT	DBS-STN / GPi (not reported)	BMT	6	UPDRS-I, UPDRS-II (ON), UPDRS-III (ON/OFF), UPDRS-IV, PDQ-39, LED, SAE
Williams et al. [33]		366	RCT	DBS-STN / GPi (not reported)	BMT	12	UPDRS-I, UPDRS-II (ON/OFF), UPDRS-III (ON/OFF), UPDRS-IV, PDQ-39, SAE

**Table 3:** Patient Characteristics If standard deviation was not reported, it was calculated from standard errors, confidence intervals, range, or other statistical indices; “Off” refers to the patients’ being off medication. H-Y: Hoehn and Yahr stage (measure of Parkinson’s disease severity; range 0–5, higher scores indicate more severity); LED (mg): levodopa equivalent dose (milligrams); N/A: not applicable, due to not being reported; SD: standard deviation

		59.0 (10.2)	15 (79)	8.5 (4.4)	1,201 (690)	N/A
Deuschl et al. [34]		60.8 (7.8)	50 (64)	13.8 (5.6)	1,175 (461)	3.8
Li et al. [32]		56.2 (9.5)	37 (58)	9.3 (3.7)	967 (528)	3.3
Okun et al. [37]		60.3 (8.3)	84 (62)	12.0 (4.7)	1,349 (712)	3.0
Schuepbach et al. [35]		52.5 (6.4)	179 (71)	7.5 (2.9)	943 (415)	< 2.5
Schüpbach et al. [36]		48.5 (3.2)	12 (60)	6.8 (1.2)	N/A	< 3
Weaver et al. [38]		62.4 (8.9)	208 (82)	11.8 (5.5)	N/A	3.4
Williams et al. [33]		59.0 (N/A)	260 (71)	11.4 (N/A)	N/A	3.4

Excluded studies

In total, 64 full-text articles were excluded (Figure 1) as they did not match the inclusion criteria. Four of them did not obviously violate the inclusion criteria but ultimately were not suitable for inclusion in this review. In short, Rothlind et al. [39] and Tramontana et al. [40] only reported neuropsychological changes as outcomes, while the study participants in Witt et al. [41] were a subset of those in Deuschl et al. [34], which was included in the meta-analysis. Finally, Charles et al. [42] was a pilot trial investigating preliminary safety and tolerability of DBS in PD and was thus inappropriately designed to address this review’s primary or secondary outcomes.

Risk of bias in included studies

**Figure 2:**
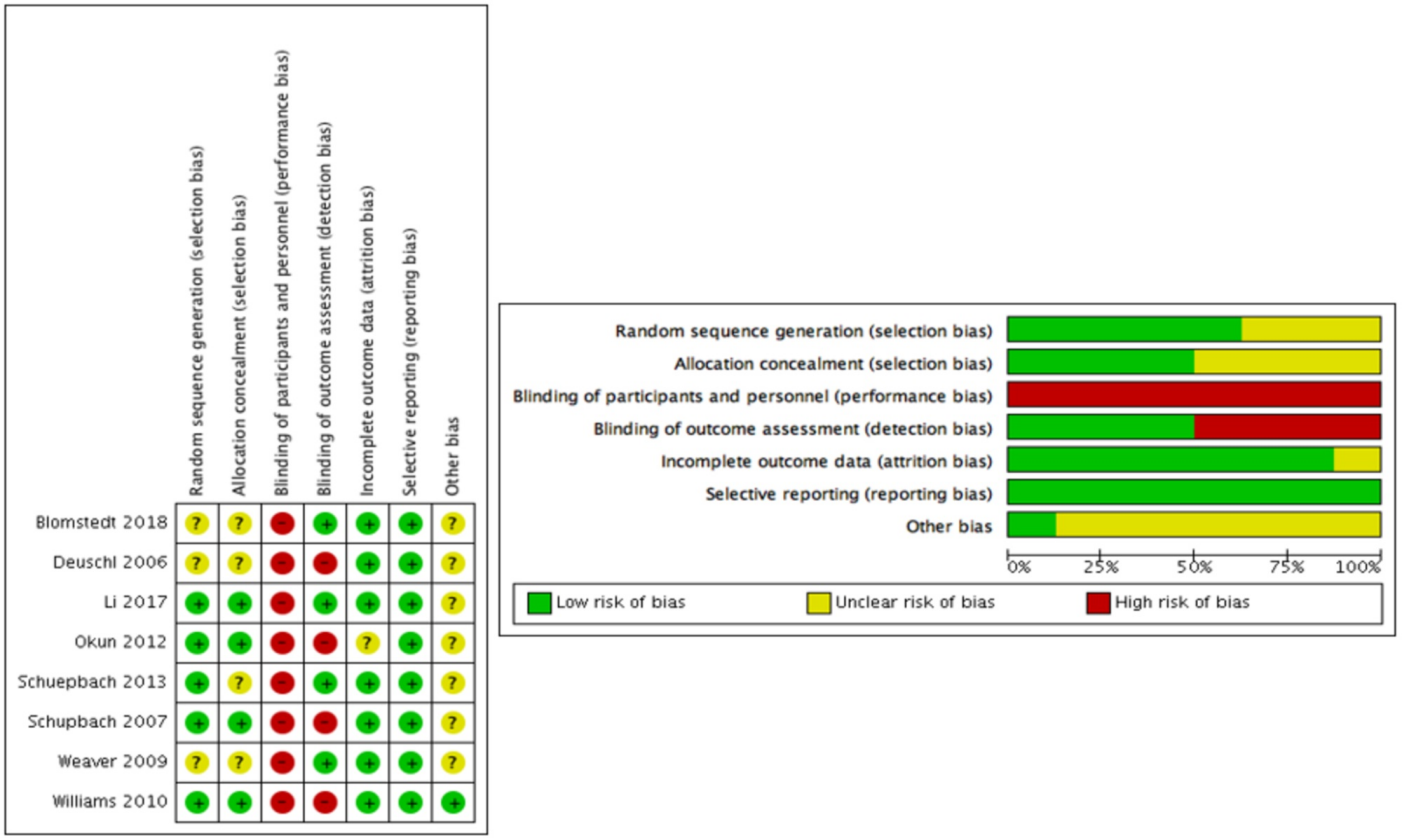
Risk of bias assessment at study level (left table) and domain level (right table) Low, unclear, and high scores given for the seven parameters assessed represented by green, yellow, and red circles, respectively.

Information on risk of bias at the study and domain level is provided in Figure 2. In terms of selection bias, the random sequence generation procedure was explicitly reported in five out of the eight studies, which were rated at low risk of bias, whereas allocation concealment was described in four studies, which were rated at low risk of bias. All included studies were rated at high risk of performance bias, as participants and personnel were unblinded. Four studies were rated at low risk of detection bias, as raters were masked to motor function assessments. Seven out of the eight studies were rated at low risk of attrition bias. One study, Okun et al. [37], was rated at unclear risk, as no explanation was provided regarding why some patients were not evaluated or included in the analysis of the UPDRS-Total, I, II (on), and IV scores. All included studies were rated at low risk of reporting bias, as all of the study’s pre-specified outcomes that were of interest in the review had been reported in a pre-specified way. Finally, seven studies were rated at unclear risk of other potential sources of bias, and in all cases, this was due to potential industry sponsorship bias. Four out of these studies were funded by the companies that developed and marketed the DBS systems. In six of the seven studies, authors reported receiving fees, honoraria, or research grants from the companies that provided the DBS systems, while the sponsors participated in the study design in two of them.

Effects of interventions

Primary Outcomes - UPDRS

**Figure 3:**
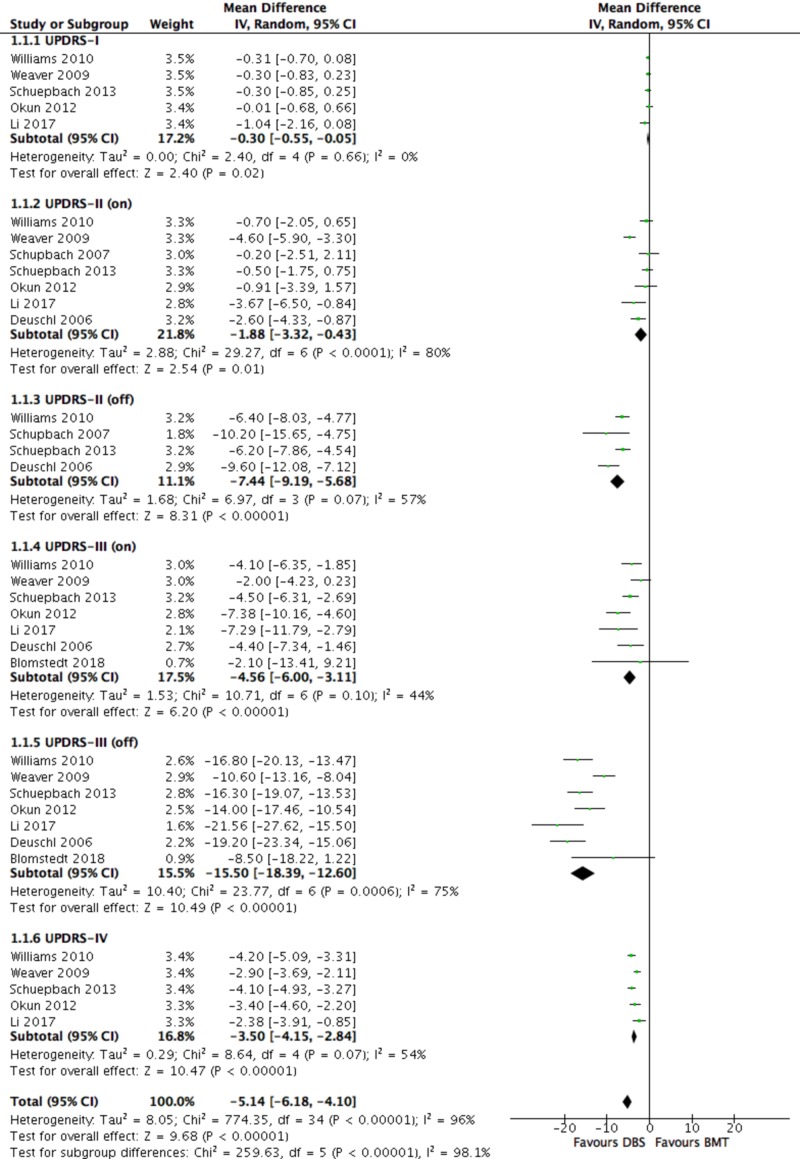
Forest plot for primary outcome analysis on Unified Parkinson’s Disease Rating Scale (UPDRS) I-IV UPDRS score ranges: I: 0–16; II: 0–52; III: 0–108; IV: 0–23; total (I–IV): 0–199 (high scores = worse clinical assessment of the patient’s Parkinson’s disease). UPDRS negative change = improvement. Mean differences in UPDRS scores along the *x*-axis, with green squares representing effect estimates and lines through them representing 95% CIs. The percentage weight is listed next to each study. Data on heterogeneity are shown at the bottom left of each score, with the relevant measure being the I^2^ score. The black diamond represents the overall effect measure. BMT: best medical therapy; CIs: confidence intervals; DBS: deep brain stimulation; df: degrees of freedom; I-V: inverse variance; “on/off”: on or off medication; Random: random effects model

Total UPDRS was significantly improved in the DBS compared to the BMT group (-5.14 (-6.18, -4.10); P < 0.00001), as was UPDRS-I (-0.30, (-0.55, -0.055); P = 0.02), UPDRS-II “on” (-1.88 (-3.32, -0.43); P = 0.01) and “off” (-7.44 (-9.19, -5.68); P < 0.00001), UPDRS-III “on” (-4.56 (-6.00, -3.11); P < 0.00001) and “off” (-15.50 (-18.39, -12.60); P < 0.00001), and UPDRS-IV (-3.50 (-4.15, -2.84); P < 0.00001) (Figure 3).

Primary Outcomes - PDQ-39

**Figure 4:**
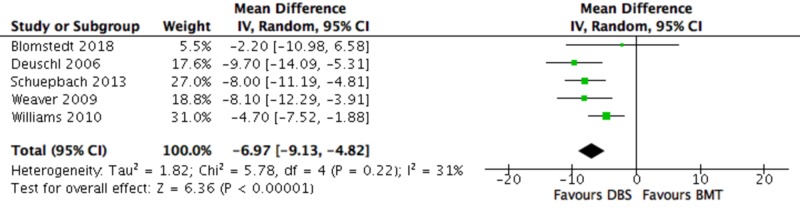
Forest plot for primary outcome analysis on health-related quality of life, as assessed by the PDQ-39 The PDQ-39 range is 0–100; the higher the score, the worse the self-reported quality of life; negative change = improvement. Mean differences in PDQ-39 scores along the x-axis, with green squares representing effect estimates and lines through them representing 95% CIs. The percentage weight is listed next to each study. Data on heterogeneity are shown at the bottom left, with the relevant measure being the I^2^ score. The black diamond represents the overall effect measure. BMT: best medical therapy; CI: confidence interval; DBS: deep brain stimulation; df: degrees of freedom; I-V: inverse variance; PDQ-39: Parkinson’s disease questionnaire; Random: random effects model

PDQ-39 was significantly improved in favour of DBS compared to the BMT group (-6.97 (-9.13, -4.82); P < 0.00001) (Figure 4).

Primary Outcomes - LED

**Figure 5:**
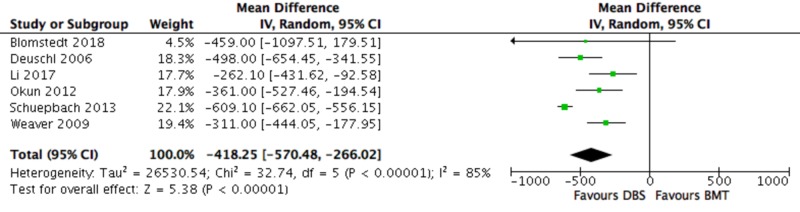
Forest plot for primary outcome analysis on LED Mean differences in LED scores, reported in milligrams (mg), along the *x*-axis, with green squares representing effect estimates and lines through them representing 95% CIs. The percentage weight is listed next to each study. Data on heterogeneity are shown at the bottom left, with the relevant measure being the I^2^ score. The black diamond represents the overall effect measure. BMT: best medical therapy; CI: confidence interval; DBS: deep brain stimulation; df: degrees of freedom; IV: inverse variance; LED: levodopa equivalent dose; Random: random effects model

LED was significantly decreased in the DBS group compared to the BMT group (-418.25, (-570.48, -266.02); P < 0.00001) (Figure 5).

Secondary Outcomes - Patients with SAE

**Figure 6:**
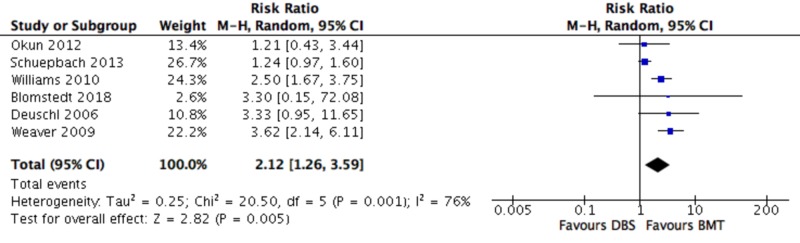
Forest plot for secondary outcome analysis on patients with serious adverse events Risk ratio for a patient experiencing a serious adverse event, along the *x*-axis, with blue squares representing effect estimates and lines through them representing 95% CIs. The percentage weight is listed next to each study. Data on heterogeneity are shown at the bottom left, with the relevant measure being the I^2^ score. The black diamond represents the overall effect measure. BMT: best medical therapy; CI: confidence interval; DBS: deep brain stimulation; df: degrees of freedom; M-H: Mantel-Haenszel

The risk of a patient experiencing an SAE was significantly higher in the DBS group compared to the BMT group (2.12 (1.26, 3.59); P = 0.005) (Figure 6).

Secondary Outcomes - Total Number of SAE

The total number of SAE was 332 vs 186 for the DBS vs BMT groups, respectively. SAEs were subcategorised to death (9 vs 3), surgery or device-related (90 vs 2), DBS-specific (69 vs 0), PD and drug-related (100 vs 134), and unrelated/other (64 vs 47) for the DBS vs BMT groups, respectively. SAEs were found to be non-parametric; thus, the Mann-Whitney U test was used for the comparison, which revealed that the total number of SAEs was significantly higher in the DBS group compared to the BMT group (P < 0.00188).

Investigation of heterogeneity

Subgroup Analysis: Early vs Advanced PD

A total of two studies [35-36] (n = 271) recruited patients with early-stage PD but data from both of them were only extracted for UPDRS-II (on) and UPDRS-II (off). The remaining six studies (n = 918) recruited patients with advanced PD.

There was no evidence of an effect of PD stage on any UPDRS (I-IV) score (P > 0.05 for all outcomes) (Figure 3). There was also no evidence of an effect of PD stage on the PDQ-39 score (test for subgroup differences: P = 0.54). LED was the only outcome where there was a statistically significant difference between the two subgroups. LED was significantly improved in the early compared to the advanced PD group (test for subgroup differences: P < 0.00001).

Within the early PD subgroup, heterogeneity was reduced to below significant levels (I^2^ < 50%), where applicable (UPDRS-II (on)/(off)). Within the advanced PD subgroup, heterogeneity was reduced to non-significant levels for LED but remained significant for UPDRS II-IV and non-significant for UPDRS-I and PDQ-39.

Sensitivity Analysis

Outcome effect sizes were not significantly affected by the exclusion of any study. In terms of the remaining heterogeneity within the advanced PD subgroup, sensitivity analysis revealed that heterogeneity was reduced to non-significant levels for UPDRS-II (on) (I^2 ^= 79% to 44%), UPDRS-III (on) (I^2 ^= 53% to 21% or 14%), and UPDRS-IV (I^2 ^= 52% to 0%), after excluding Weaver et al. [38], Okun et al. [37], or Weaver et al. [38], and Williams et al. [33], respectively. Regarding secondary outcomes, excluding Schuepbach et al. [35] from the meta-analysis assessing the relative risk of a patient experiencing an SAE in the DBS and BMT groups reduced heterogeneity from I^2 ^= 76% to 0%.

Summary of main results

This systematic review and meta-analysis focused on evaluating the efficacy and safety of DBS in the treatment of PD in comparison to BMT. Eight RCTs with a total of 1,189 participants were identified. Overall, DBS was found to be superior in efficacy and inferior in safety to BMT, as was hypothesized. In addition, DBS may have a similar efficacy in early and advanced stage PD, with the exception of LED, where there was a greater improvement in patients with early PD.

Primary outcomes

Specifically, in terms of efficacy, there was a significant improvement in all primary outcomes (UPDRS, PDQ-39, LED) in the DBS vs the BMT group. Total UPDRS, as well as every UPDRS sub-score (I-IV), were significantly improved in the DBS vs the BMT group in both “on” and “off” states, meaning that DBS was superior to BMT in improving the mental status, behaviour, mood (UPDRS-I), ADLs (UPDRS-II), motor function (UPDRS-III), and complications from therapy (UPDRS-IV) in PD patients. Importantly, the largest improvements were seen in the patients’ motor function (MD -15.50), the primary treatment goal for PD patients, followed by ADLs (MD -7.44), in the “off” medication state; greater benefits were expected for DBS vs BMT in the “off” state, due to the likelihood of a ceiling effect in the ”on” state, although this represents an artificial situation (no medication withdrawal in real life). Similarly, the patients’ QoL, as reflected in the reduction in PDQ-39 (MD -6.97) and medication doses (LED) (MD -418.25), were significantly improved in the DBS vs BMT groups. Another notable observation is that DBS was superior to BMT in every single aforementioned outcome in all included studies. However, the fact that only three out of eight RCTs [34-35, 38] explicitly referred to the optimization details of the medical therapy means that the validity of “BMT” as control could be questioned, as it is unclear if the “best” medical therapy was indeed provided to the patients in the control groups. Potentially, an overestimation of the benefit of DBS over true BMT may have thus occurred.

Regarding the subgroup analysis, no evidence of different treatment effect was found between early vs advanced PD in the UPDRS and PDQ-39 scores. However, a greater effect was observed on the LED in the early vs advanced PD group (P < 0.00001). In Schuepbach et al. [35], i.e., the only study measuring LED in early PD patients, there was a 25% increase in LED during follow-up in the control group, which is not surprising, given that younger patients with lower disease severity were recruited; this, coupled with the decrease in LED in the DBS group, could explain why a significant difference was found between the two groups. Moreover, in the advanced PD subgroup, the smallest reductions of LED in patients receiving DBS were seen in Weaver et al. [38] and Blomstedt et al. [31]. This could be because half of the experimental group in Weaver et al. [38] received DBS in the GPi, which has been shown to have a smaller effect on LED than STN [43], while patients in Blomstedt et al. [31] received DBS in the cZi, which, though still unknown, could also be inferior to STN in LED reduction. Another reason for the non-significant findings could be the fact that the disease severity of the patients between the two subgroups was not significantly different, evident from the relatively similar H-Y stage scores. Also, patients in the Blomstedt et al. [31] and Li et al. [32] studies had an approximately similar mean disease duration to the patients in the two early PD studies [35-36].

Secondary outcomes

In terms of safety, DBS was found to be inferior to BMT for both secondary outcomes. The risk of a patient experiencing an SAE in the DBS group was more than double that of the BMT group (RR 2.12) and that result was consistent across all studies. Furthermore, there was a significantly higher number of SAEs in the DBS vs the BMT group (332 vs 186; P < 0.00188), with almost half (159/332) of the SAEs in the DBS group being related to the surgery, device, or stimulation therapy. Of the nine deaths in the DBS group, three were due to intraoperative cerebral haematoma and three were due to suicide, whereas of the three deaths in the BMT group, one was due to suicide and one was due to an accident during a psychotic episode, with the rest being unrelated to treatment. Nevertheless, it is unlikely that the classification of the SAE into a particular category was clear-cut, so that these numbers may be misleading. For instance, some SAEs classified as DBS-specific could have also perhaps been classified as PD and drug-related. Notably, psychosis was deemed to be DBS-specific, but neuropsychiatric disturbances were deemed to be PD and drug-related. Some studies also classified some SAEs as “stimulation or medication-related”, making it difficult to differentiate which treatment was responsible for these events, since patients in the DBS group were also on medication. Moreover, more “unrelated/other” SAEs were observed in the DBS vs the BMT group (64 vs 47), which overestimates the significance of the difference (P-value) in the number of SAEs between the two groups. Another reason to cautiously interpret these results is that the patients’ mental status, behaviour, and mood (UPDRS-I), complications from therapy (UPDRS-IV), and overall QoL (PDQ-39) - all linked to tolerability/acceptability of therapy - were significantly improved in the DBS vs the BMT group. However, this may at least partly be explained by a relative reduction in non-serious adverse events in the DBS group, the analysis of which, however, was beyond the scope of this review.

Completeness and applicability of evidence

Although the number of RCTs in this review was small, the total sample size was relatively large (n = 1,189). However, each outcome was measured using data from four (UPDRS-II “on”) to seven (UPDRS-III “on” or “off” and UPDRS-II “on”) studies, therefore, suggesting a comparatively greater completeness of evidence in motor control and ADLs. This was even more pronounced in the subgroup analysis where two studies were included in the early PD group, and in fact, data from only one study were available for all outcomes, except UPDRS-II “on” and “off”. It would, therefore, be rather inaccurate to generalise the conclusions from this analysis. Moreover, follow-up was 12 months or less in six out of the eight studies, which creates uncertainty about the long-term viability of the efficacy outcomes and introduces bias to the incidence of SAEs, some of which might not appear until later on. For example, Volkmann et al. [44] showed that motor symptoms returned back to baseline five years after GPi-DBS surgery, although Krack et al. [45] showed that motor symptoms continuously improved five years after STN-DBS surgery. This difference might be attributed to the different DBS target areas, but Weaver et al. [46] demonstrated that improvement of motor symptoms remained stable over three years and did not differ by the surgical target. Furthermore, exclusion criteria regarding psychiatric co-morbidities and lack of preoperative levodopa responsiveness, which has been found to be predictive of improvement in UPDRS scores following DBS surgery [47], meant that similar benefits may not be realised by a sizeable proportion of the target population. It is also unlikely that patients in real life practice receive medical therapy as optimized as in the RCTs, which might underestimate the true relative effectiveness of DBS over medication.

Quality of the evidence

Despite the robust design of the included studies (parallel-group RCTs), blinded outcome assessments were only performed in four studies, whereas no blinding of participants or personnel was performed in any study. Sham surgery on patients in the control group (i.e., to insert electrodes and stimulators but not switch them on) could overcome this, but it would not have been practical (e.g., higher cost) while raising ethical considerations. Furthermore, masking attempts may have been ineffective, as patients would have been able to tell if their stimulator was switched on (placebo effect). There was evidence of unclear risk of selection bias, as random sequence generation and allocation concealment were not explicitly reported in three and four studies, respectively. No risk of reporting bias and a minor risk of attrition bias (unclear risk in one study) were found. Another potential source of bias was industry bias, present in seven studies, as the DBS equipment provider sponsored the studies (Figure 2). Hence, heterogeneity regarding methodologic quality was clearly present. Additional sources of heterogeneity were sample size, follow-up time, DBS target area, DBS settings, and disease duration/severity. The subgroup analysis only removed the heterogeneity observed in LED. The remaining heterogeneity was explained by the sensitivity analysis, which showed that Weaver et al. [37] was mostly responsible for the observed heterogeneity, as half the experimental group received DBS in the GPi, the mean age of participants was higher (62.4), and was one out of three studies that performed blinded motor evaluations, which may have accounted for the smaller improvement in UPDRS-III scores. Also, definitions of SAE vs non-serious adverse effect and their subsequent classification to categories varied amongst RCTs. The heterogeneity in SAE was removed after excluding Schuepbach et al. [35], where it was especially unclear whether DBS or medication accounted for some SAEs. Finally, neither the DBS procedures or BMT guidelines were standardized across RCTs, creating more heterogeneity. Nevertheless, random-effect models were used in the statistical analysis to account for this heterogeneity.

Bias in the review process

It is possible that publication bias could have affected the results. However, according to the Cochrane Handbook for Systematic Reviews of Interventions [24], methods for detecting publication bias, such as funnel plots, are not accurate when < 10 studies are included and, therefore, such methods have been avoided. In addition, too few studies were included in the subgroup analysis, which makes the comparison unreliable. The conversion of non-normally-distributed statistics (median (range)) to normally-distributed statistics (mean (SD)) may be another source of bias in the analysis. This was evident from the wide SD of all outcomes from Blomstedt et al. [32], where such methods were utilised, although this study was consistently given the least weight to account for this. Finally, only studies published in English were included, which could also create bias.

Agreements/disagreements with other reviews

The results for the primary outcomes and their effect sizes were similar to those reported in previous reviews, e.g., in Perestelo-Pérez et al. [16] and Xie et al. [18], where DBS was found to significantly improve UPDRS, PDQ-39, and LED in patients with PD compared to BMT. However, Perestelo-Pérez et al. [18] reported estimates as standardized MD due to the use of various scales for outcome assessments, while Xie et al. [18] extracted data as final values rather than change from baseline, which may compromise the precision of the results. Previous meta-analyses comparing either DBS in early vs advanced stage PD or SAEs in patients receiving DBS vs BMT are not known to have been published.  

## Conclusions

Substantial evidence to support the use of DBS in favour of BMT in the treatment of PD was found, given the observed improvements in motor control, functionality, and QoL. Moreover, the reduced LED may lead to lower levodopa-related adverse effects and higher compliance while also significantly decreasing medication costs, which may offset the expense of the DBS procedure in the long term. These benefits, however, need to be carefully weighed against the higher risk of SAEs, a large proportion of which were surgery-related, stressing the importance of an experienced surgical team performing the procedure.

The efficacy of DBS has been established, yet the question remains as to whether DBS should be considered as a treatment option at an earlier stage than the current recommendations. Despite the promising results of the subgroup analysis, its limitations did not allow for conclusions to be drawn with a high degree of confidence. Further RCTs are required to evaluate the optimum time window for DBS to achieve maximum benefits. Additionally, a unified approach to assess the severity and cause of SAEs is required from future RCTs in order to remove bias in subsequent meta-analytic estimates. Finally, and perhaps most crucially, enhanced understanding of the mechanism of DBS in relation to the pathophysiology of the basal ganglia is needed to provide detailed explanations of the observed therapeutic benefits in patients with PD and identify prognostic factors that may predict a positive outcome to DBS.

## References

[1] Tysnes OB, Storstein A (2017). Epidemiology of Parkinson’s disease. J Neural Transm (Vienna).

[2] Jankovic J (2008). Parkinson’s disease: clinical features and diagnosis. J Neurol Neurosurg Psychiatry.

[3] Chaudhuri KR, Schapira AH (2009). Non-motor symptoms of Parkinson’s disease: dopaminergic pathophysiology and treatment. Lancet Neurol.

[4] Magrinelli F, Picelli A, Tocco P (2019). Pathophysiology of motor dysfunction in Parkinson’s disease as the rationale for drug treatment and rehabilitation. Parkinsons Dis.

[5] Crosby NJ, Deane K, Clarke CE (2019). Amantadine in Parkinson’s disease. Cochrane Database Syst Rev.

[6] Jankovic J (2019). Complications and limitations of drug therapy for Parkinson’s disease. Neurology.

[7] Stowe R, Ives N, Clarke CE (2008). Dopamine agonist therapy in early Parkinson’s disease. Cochrane Database Syst Rev.

[8] Li Q, Qian ZM, Arbuthnott GW (2014). Cortical effects of deep brain stimulation: implications for pathogenesis and treatment of Parkinson disease. JAMA Neurol.

[9] Follett KA (2000). The surgical treatment of Parkinson's disease. Annu Rev Med.

[10] Malkki H (2015). Deep brain stimulation might alleviate parkinsonism by reducing excessive synchronization in primary motor cortex. Nat Rev Neurol.

[11] Xu W, Russo GS, Hashimoto T (2008). Subthalamic nucleus stimulation modulates thalamic neuronal activity. J Neurosci.

[12] Brown P, Mazzone P, Oliviero A (2004). Effects of stimulation of the subthalamic area on oscillatory pallidal activity in Parkinson's disease. Experiment Neurol.

[13] Rubin JE, Terman D (2019). High frequency stimulation of the subthalamic nucleus eliminates pathological thalamic rhythmicity in a computational model. J Comput Neurosci.

[14] Guo Y, Rubin JE, McIntyre CC (2019). Thalamocortical relay fidelity varies across subthalamic nucleus deep brain stimulation protocols in a data-driven computational model. J Neurophysiol.

[15] Okun MS (2012). Deep-brain stimulation for Parkinson’s disease. New Engl J Med.

[16] Perestelo-Pérez L, Rivero-Santana A, Pérez-Ramos J (2014). Deep brain stimulation in Parkinson’s disease: meta-analysis of randomized controlled trials. J Neurol.

[17] Tan ZG, Zhou Q, Huang T, Jiang Y (2019). Efficacies of globus pallidus stimulation and subthalamic nucleus stimulation for advanced Parkinson’s disease: a meta-analysis of randomized controlled trials. Clin Interv Aging.

[18] Xie CL, Shao B, Chen J (2019). Effects of neurostimulation for advanced Parkinson’s disease patients on motor symptoms: a multiple-treatments meta-analysis of randomized controlled trials. Sci Rep.

[19] Zrinzo L, Foltynie T, Limousin P, Hariz MI (2019). Reducing hemorrhagic complications in functional neurosurgery: a large case series and systematic literature review. J Neurosurg.

[20] Grill WM (2005). Safety considerations for deep brain stimulation: review and analysis. Expert Rev Med Devices.

[21] Hughes AJ, Daniel SE, Kilford L, Lees AJ (1992). Accuracy of clinical diagnosis of idiopathic Parkinson’s disease: a clinico-pathological study of 100 cases. J Neurol Neurosurg Psychiatry.

[22] Martínez-Martín P, Gil-Nagel A, Gracia LM (1994). Unified Parkinson's disease rating scale characteristics and structure. Mov Disord.

[23] Marinus J, Ramaker C, van Hilten JJ, Stiggelbout AM (2002). Health related quality of life in Parkinson’s disease: a systematic review of disease specific instruments. J Neurol Neurosurg Psychiatry.

[24] Higgins JPT, Green S (2018). Cochrane Handbook for Systematic Reviews of Interventions Version 5.1.0. Online] The Cochrane Collaboration.

[25] (2018). Better systematic review management. http://www.covidence.org/home.

[26] Hoehn MM, Yahr MD (2019). Parkinsonism: onset, progression and mortality. Neurology.

[27] (2018). RevMan 5. Copenhagen: The Nordic Cochrane Centre, The Cochrane Collaboration.

[28] (2018). Social science statistics: Mann-Whitney U test calculator. Online] Available.

[29] (2018). Kirkman TW: statistics to use. http://www.physics.csbsju.edu/stats/.

[30] Sutton AJ, Abrams KR, Jones DR, Sheldon TA, Song F (2018). Methods for Meta-analysis in Medical Research. Wiley.

[31] Blomstedt P, Stenmark Persson R, Hariz GM (2018). Deep brain stimulation in the caudal zona incerta versus best medical treatment in patients with Parkinson’s disease: a randomised blinded evaluation. J Neurol Neurosurg Psychiatry.

[32] Li D, Zhang C, Gault J (2017). Remotely programmed deep brain stimulation of the bilateral subthalamic nucleus for the treatment of primary Parkinson disease: a randomized controlled trial investigating the safety and efficacy of a novel deep brain stimulation system. Stereotact Funct Neurosurg.

[33] Williams A, Gill S, Varma T (2010). Deep brain stimulation plus best medical therapy versus best medical therapy alone for advanced Parkinson's disease (PD SURG trial): a randomised, open-label trial. Lancet Neurol.

[34] Deuschl G, Schade-Brittinger C, Krack P (2006). A randomized trial of deep-brain stimulation for Parkinson’s disease. New Engl J Med.

[35] Schuepbach WMM, Rau J, Knudsen K (2013). Neurostimulation for Parkinson’s disease with early motor complications. New Engl J Med.

[36] Schüpbach WMM, Maltête D, Houeto JL (2007). Neurosurgery at an earlier stage of Parkinson disease: a randomized, controlled trial. Neurology.

[37] Okun MS, Gallo BV, Mandybur G (2012). Subthalamic deep brain stimulation with a constant-current device in Parkinson’s disease: an open-label randomised controlled trial. Lancet Neurol.

[38] Weaver FM, Follett K, Stern M (2009). Bilateral deep brain stimulation vs best medical therapy for patients with advanced Parkinson disease: a randomized controlled trial. JAMA.

[39] Rothlind JC, York MK, Carlson K (2015). Neuropsychological changes following deep brain stimulation surgery for Parkinson’s disease: comparisons of treatment at pallidal and subthalamic targets versus best medical therapy. J Neurol Neurosurg Psychiatry.

[40] Tramontana MG, Molinari AL, Konrad PE (2019). Neuropsychological effects of deep brain stimulation in subjects with early stage Parkinson’s disease in a randomized clinical trial. J Parkinsons Dis.

[41] Witt K, Daniels C, Reiff J (2019). Neuropsychological and psychiatric changes after deep brain stimulation for Parkinson’s disease: a randomised, multicentre study. Lancet Neurol.

[42] Charles D, Konrad PE, Neimat JS (2014). Subthalamic nucleus deep brain stimulation in early stage Parkinson’s disease. Parkinsonism Relat Disord.

[43] Andrade P, Carrillo-Ruiz JD, Jiménez F (2019). A systematic review of the efficacy of globus pallidus stimulation in the treatment of Parkinson’s disease. J Clin Neurosci.

[44] Volkmann J, Allert N, Voges J (2004). Long-term results of bilateral pallidal stimulation in Parkinson’s disease. Ann Neurol.

[45] Krack P, Batir A, Van Blercom N (2019). Five-year follow-up of bilateral stimulation of the subthalamic nucleus in advanced Parkinson’s disease. New Engl J Med.

[46] Weaver FM, Follett KA, Stern M (2012). Randomized trial of deep brain stimulation for Parkinson disease: thirty-six-month outcomes. Neurology.

[47] Bronstein JM, Tagliati M, Alterman RL (2011). Deep brain stimulation for Parkinson disease. Arch Neurol.

